# Robust Expression of the Human Neonatal Fc Receptor in a Truncated Soluble Form and as a Full-Length Membrane-Bound Protein in Fusion with eGFP

**DOI:** 10.1371/journal.pone.0081350

**Published:** 2013-11-18

**Authors:** Johan Seijsing, Malin Lindborg, John Löfblom, Mathias Uhlén, Torbjörn Gräslund

**Affiliations:** 1 School of Biotechnology, KTH Royal Institute of Technology, Stockholm, Sweden; 2 Affibody AB, Solna, Sweden; Cleveland Clinic Lerner Research Institute, United States of America

## Abstract

Studies on the neonatal Fc receptor (FcRn) have revealed a multitude of important functions in mammals, including protection of IgG and serum albumin (SA) from lysosomal degradation. The pharmacokinetic behavior of therapeutic antibodies, IgG-Fc- and SA-containing drugs is therefore influenced by their interaction with FcRn. Pre-clinical development of such drugs is facilitated if their interaction with FcRn can be studied *in vitro*. For this reason we have developed a robust system for production of the soluble extracellular domain of human FcRn as well as the full-length receptor as fusion to green fluorescent protein, taking advantage of a lentivirus-based gene delivery system where stable over-expressing cells are easily and rapidly generated. Production of the extracellular domain in multiple-layered culture flasks, followed by affinity purification using immobilized IgG, resulted in capture of milligram amounts of soluble receptor per liter cell culture with retained IgG binding. The receptor was further characterized by SDS-PAGE, western blotting, circular dichroism spectroscopy, ELISA, surface plasmon resonance and a temperature stability assay showing a functional and stable protein of high purity. The full-length receptor was found to be successfully over-expressed in a membrane-bound form with retained pH-dependent IgG- and SA-binding.

## Introduction

The neonatal Fc receptor (FcRn) is a heterodimeric protein consisting of a transmembrane MHC class I-like heavy chain (FcRn α-chain) and the β_2_-microglobulin (β_2_m) light chain, of which the latter is also a part of MHC class I receptors [1,2].

FcRn is predominantly located in endosomes and is able to bind to serum albumin (SA) and immunoglobulin G (IgG) at pH≤6.5 and release them at pH≥7.0 [3–5]. The receptor carries out several distinct tasks in mammals. When IgG, SA and other serum proteins are passively endocytosed by cells in contact with blood, the pH becomes gradually lower in the formed endosomes permitting binding of IgG and SA to FcRn. The receptor is then, together with its bound ligand, transported back via recycling endosomes to the plasma membrane, and thus rescues the ligand from lysosomal degradation. After returning to the plasma membrane, the pH increases above 7 at which point the bound ligand is released. The pH dependent binding between IgG and FcRn does not seem to rely on any large structural changes in the proteins, but instead from protonation or deprotonation of histidines in the hinge region between CH2 and CH 3 in Fc and corresponding acidic amino acids in FcRn [6–8]. The interaction between FcRn and SA is less characterized, but is at least partly dependent on His-166 in FcRn [9]. The IgG and SA sites of interaction on FcRn are distinct and it has been shown that the three proteins may form a ternary complex [10]. In humans, FcRn is involved in transmission of IgG from the mother to the fetus via the placenta [11] and in rodents for transmission of IgG from the mother’s milk to the newborn via the proximal small intestine [1].

The expression pattern of FcRn differs between species, but it is often widely expressed on cells in the blood brain barrier, upper airway epithelium, kidneys, vascular endothelia, professional antigen presenting cells as well as other cells of hematopoietic origin [12]. It has long been thought that the vascular endothelium was the major site of IgG recycling, but recently it was shown that also cells of hematopoietic origin contribute to the FcRn-mediated half-life extension of IgG [13,14].

In recent years, efforts have been undertaken to modulate the FcRn interaction of therapeutic antibodies and to develop IgG-Fc- or SA-containing drugs, with the aim to change their pharmacokinetic behavior [15]. Such efforts would be facilitated by a plentiful source of high quality FcRn to be used for *in vitro* experiments. In this study, the goal has been to develop a robust expression system for human FcRn in a truncated soluble form and as a full-length membrane-bound protein in fusion with eGFP.

The extracellular domain of FcRn (FcRn_ECD_) from different species has been recombinantly expressed in many different organisms. Although both gram negative bacteria [16] and yeast [17] have been used, it is more common to utilize cells from higher eukaryotes such as CHO cells [18], different derivates of 293T cells [19-21] and High-5 insect cells [22]. In mammalian cells, most commonly a plasmid is introduced into the cells giving transient expression.

Purification of FcRn_ECD_ has mostly been performed by immobilized metal-ion affinity chromatography (IMAC) on a receptor that has been extended by a hexa-histidine tag [16,17,20–23]. It has also been shown that the FcRn_ECD_ may be recovered by affinity chromatography where immobilized IgG is used as the ligand on the column [18,24]. In these studies the inherent pH dependent binding to IgG is utilized, where FcRn is captured on the column at a pH<6.5 and eluted by raising the pH above 7. In contrast to IMAC purification, this strategy only recovers receptor molecules with intact IgG binding.

Full-length FcRn may be expressed as a fusion to a fluorescent protein to allow for example receptor tracking in live cells. A transient system has previously been described where co-transfection of a plasmid encoding the FcRn α-chain as fusion to green fluorescent protein and a plasmid encoding β_2_m resulted in successful expression of functional full-length FcRn [25,26].

One of the main differences in FcRn produced in different host cells is the glycosylation pattern. Even though the interaction between FcRn and its ligands IgG and SA appears to be unaffected by the glycosylation pattern [16], it has been shown to modulate FcRn transport in the cell [27]. Therefore, we reasoned that the best host for production would be human cells. Thus, FcRn_ECD_ was expressed in the SKOV-3 cell line, and the full-length FcRn as a fusion to eGFP in the HeLa cell line. In both cases, the genes were introduced using lentivirus-derived vectors, allowing for facile and quick selection for double integrants by two different selective agents. For FcRn_ECD_, we used the intrinsic property of pH dependent binding to IgG to recover active protein of very high purity in a single affinity chromatographic purification step from the culture medium. Both forms of the protein were characterized biochemically.

## Materials and Methods

### Materials

PCR primers were from Eurofins MWG Operon (Ebersberg, Germany). Chemicals and cell culture reagents were from SigmaAldrich (St. Louis, MO, USA) unless otherwise stated.

### Vector constructions

**Figure 1:**
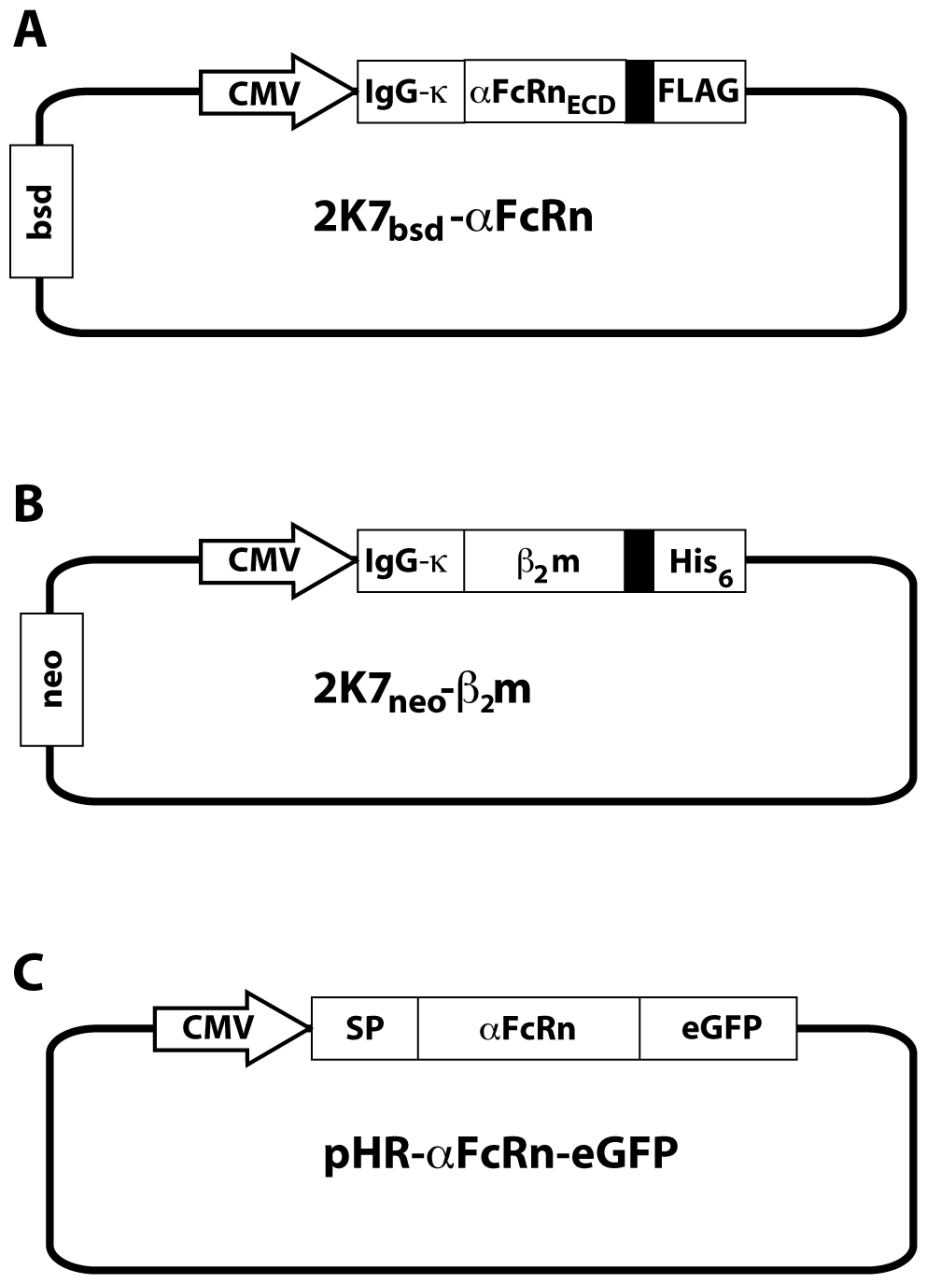
Vector design. Schematic description of the plasmids used in the study. CMV, cytomegalovirus promoter; Ig-κ, Ig-κ chain leader sequence; filled black boxes, GGGS-spacer. (A) The expression plasmid for the extracellular domain of FcRn: αFcRn_ECD_, the extracellular domain of FcRn; FLAG, the FLAG-tag; bsd, blasticidin resistance gene. (B) The expression plasmid for human β_2_m: His_6_, hexa-histidine tag; neo, G418 sulfate resistance gene. (C) The expression plasmid for full-length FcRn as a fusion to enhanced green fluorescent protein (eGFP) including the endogenous signal peptide of FcRn α-chain (SP).

The genes encoding human FcRn α-chain (Genebank BC008734.2) and human β_2_m (Genebank BC032589.1) were obtained from OpenBiosystems (Huntsville, AL, USA). Using PCR overlap extension, amino acid 24-290 of human FcRn α-chain was amplified to a construct consisting of attB1-site/Kozak sequence/Ig-κ chain leader sequence/FcRn_ECD_/GS-linker/FLAG-tag/attB2 site, and amino acid 20-119 of human β_2_m to attB1-site/Kozak sequence/Ig-κ-chain leader sequence/β_2_m/GS-linker/His_6_-tag/attB2-site. The constructs were inserted into the plasmid pDONOR221 by recombination using the Gateway system (Invitrogen, CA, USA) according to the manufacturer’s instruction. After verification of correct sequences by DNA sequencing, the human FcRn_ECD_ α-chain construct was inserted into 2K7_bsd_ [28] using multi-site gateway cloning together with the promoter-containing plasmid pENTR-CMV [29] resulting in the vector 2K7_bsd_-αFcRn_ECD_ (Figure 1A). The human β_2_m construct was similarly inserted into 2K7_neo_ [28] giving the vector 2K7_neo_-β_2_m (Figure 1B).

The full-length gene encoding human FcRn α-chain (amino acids 1-365) was amplified by PCR to yield an amplicon with a *Bcl*I restriction site and Kozak sequence in the 5’-end, and an *Mlu*I restriction site in the 3’-end. It was subsequently inserted into pHR-cPPT-CMV-eGFP [30] using *Bcl*I and *Mlu*I. The construct was verified by DNA sequencing and denoted pHR-αFcRn-eGFP (Figure 1C).

### Cell culture

The HEK293T, SKOV-3 and HeLa cell lines were obtained from ATCC (via LGC Standards, Borås, Sweden). Cells were grown at 37°C in a humidified incubator in the presence of 5% CO_2_. Complete medium for the HEK293T cell line was Dulbecco’s modified eagle medium supplemented with 10% fetal bovine serum (FBS), 1% Antibiotic Antimycotic Solution (AA) and 1% MEM Non-essential Amino Acid Solution. Complete medium for the SKOV-3 cell line was McCoys 5A medium supplemented with 10% FBS and 1% AA. Complete medium for the HeLa cell line was Eagle's Minimum Essential Medium supplemented with 10% FBS and 1% AA. 

### Preparation of recombinant lentiviral vectors and gene insertions in SKOV-3 and HeLa cell lines

The plasmids 2K7_bsd_-αFcRn_ECD_, 2K7_neo_-β_2_m or pHR-αFcRn-eGFP were co-transfected with VSV-G envelope plasmid and gag/pol packaging plasmid into HEK293T cells using calcium chloride transfection [31,32]. HEK293T culture supernatants containing formed lentiviral particles were clarified by centrifugation and filtration. Lentivirus encoding αFcRn_ECD_ and β_2_m were used to sequentially transduce SKOV-3 cells. Successful double integrants were selected by addition of blasticidin and G418 sulfate (Invitrogen) to the culture medium while passaging the cells for two weeks. The resulting stably transduced SKOV-3 cell line was denoted SKOV-3-αFcRn_ECD_/β_2_m. HeLa cells were transduced with the lentiviral vectors carrying the genes encoding αFcRn-eGFP and human β_2_m. The stably transduced HeLa cell line was denoted HeLa-αFcRn-eGFP/β_2_m.

### Expression and purification of recombinant FcRn_ECD_

SKOV-3-αFcRn_ECD_/β_2_m cells were expanded and 1.5*10^7^ cells were seeded in a HYPERFlask (Corning, NY, USA) in 560 ml complete growth medium. Five days later the medium was changed to complete growth medium without FBS and protein production was carried out for an additional 120 h after which the supernatant was collected, passed through a 45 µm filter and frozen at -80°C.

Protein purification was carried out on an FPLC-ÄKTA explorer-system (Amersham Pharmacia Biotech, Uppsala, Sweden). Human IgG (Pharmacia, Uppsala, Sweden) at a concentration of 10 mg/ml in 0.2 M NaHCO_3_, 0.5 M NaCl (pH 8.3) was coupled to a 1 ml HiTrap NHS-activated HP column (GE Healthcare, Uppsala, Sweden) according to manufacturer’s instruction. The SKOV-3-αFcRn_ECD_/β_2_m supernatant was thawed and the pH was adjusted to 5.8. The supernatant was subsequently loaded in batches of 100 ml onto the column that had previously been equilibrated with running buffer (20 mM Bis-Tris, pH 5.8). The column was washed with 20 ml running buffer and eluted using 50 mM Tris, pH 8.1.

### SDS-PAGE and Western blot

SDS-PAGE analysis on eluted fractions from the protein purification was carried out using a 4–12% Bis-Tris Gel (Invitrogen). After electrophoresis, the gel was stained with GelCode Blue Stain Reagent (Pierce, IL, USA). The gel was documented and subsequently stained by SilverXpress® Silver Staining Kit (Invitrogen). Western blotting was carried out by transfer of material separated on a 4–12% Bis-Tris gel to an Amersham Hybond™-C Extra (GE Healthcare) nitrocellulose membrane. The membrane was blocked with 5% non-fat dry milk (Semper, Falun, Sweden) in TBS+T (50 mM Trizma base, 150 mM NaCl, 0.05% Tween-20, pH 8.0) for 1 h, after which the membrane was probed with a mixture of rabbit α-FCGRT polyclonal antibody (Atlas Antibodies, Stockholm, Sweden) at a concentration of 0.15 µg/ml and rabbit α-β_2_m polyclonal antibody (Atlas Antibodies) at a concentration of 0.23 µg/ml in TBS+T. The membrane was subsequently incubated with stabilized goat α-rabbit antibody conjugated with horseradish peroxidase (HRP) (Pierce), diluted 1:10000 in TBS-T. After addition of TMB Substrate (Pierce) an image of the membrane was acquired on Amersham Hyperfilm ECL (GE Healthcare). 

### Secondary structure determination

Circular dichroism was measured using a J-810 Spectropolarimeter (Jasco Corporation, MD, USA) at 20°C from 260 to 200 nm. FcRn was at a concentration of 300 µg/ml in PBS. The secondary structure content of the spectrum was determined using CDNN 2.1 software (Applied Photophysics, Leatherhead, United Kingdom). 

#### ELISA

Unless otherwise noted, the steps in ELISAs were performed at r.t. To investigate the pH dependent binding of FcRn and hIgG, an ELISA was performed. Human FcRn_ECD_ was biotinylated according to the manufacturer’s instruction (Thermo scientific, MA, USA). Human IgG (Pharmacia) was diluted to a concentration of 10 µg/ml and was added to a half well ELISA plate. The plate was incubated 14 h at 4°C after which it was rinsed with water. The plate was blocked by addition of PBS of respective pH from 5.5-8.0 containing 0.5% casein (PBSC) for 1.5 hours. After removing blocking solution, 50 µl of biotinylated human FcRn_ECD_ diluted to 0.5 µg/ml in PBSC of respective pH from 5.5-8.0 was added to the wells followed by incubation for 1 h. The wells were washed with PBST of respective pH. HRP-conjugated Streptavidin (Thermo Scientific) was added to each well followed by incubation for 30 min. The plate was developed by TMB substrate (Pierce), and the reaction was stopped using 2 M H_2_SO_4_. The absorbance of each well was measured at 450 nm.

The ELISAs to investigate the functionality of the FLAG- and His_6_-tags were performed as follows. Human IgG was diluted in 0.2 M carbonate buffer (pH 9.6) to a concentration of 10 µg/ml, and immobilized to the bottom of a half well ELISA plate for 48 h at 4°C. The plate was blocked with PBSC at pH 6.0 for 4 h. After washing, FcRn_ECD_ diluted to 2 µg/ml in PBSC was added to the wells followed by incubation for 1 h. The wells were washed and HRP-conjugated goat α-FLAG antibody (Abcam) at 0.1 µg/ml or HRP-conjugated goat α-His_6_ antibody (Abcam) at 0.1µg/ml in PBSC was added to the plate and incubated for 1 h. The wells were washed and developed by TMB substrate (Pierce). 

### Biosensor analysis

The interaction between human FcRn and IgG or SA was measured by biosensor analysis using a Biacore 3000 instrument (GE Healthcare). FcRn_ECD_ at a concentration of 7.5 µg/ml was immobilized on a carboxymethylated dextran chip with amine coupling chemistry at pH 4.65. The surface used for pH dependent binding and kinetic analysis of the HSA/FcRn_ECD_ interaction had an immobilization level of 590 RU. The surface used for kinetic analysis of the Bevacizumab/FcRn_ECD_ interaction had an immobilization level of 315 RU. A reference flow cell was created by activation and deactivation, and was subtracted from the response from the FcRn_ECD_ flow cell. McIlvaines phosphate-citrate buffer with 0.005% tween at pH 6.0 or 7.4 was used as running buffer, and for dilution of the analytes. Binding of Bevacizumab (Roche, Basel, Switzerland), 1-D1K (Mabtech, Stockholm, Sweden) and HSA (Novozymes, Bagsvaerd, Denmark) was investigated at a flow-rate of 50 µl/min at 25°C. 

### Fluorescence microscopy

Glass slides were coated with fibronectin for 1 h followed by plating of 15000 HeLa-αFcRn-eGFP/β_2_m cells per well. After attachment for 3 h the cells were washed, fixated with cold 4% paraformaldehyde in growth media, permeabilized using 0.1% Triton X-100 (SigmaAldrich) in PBS and stained with 300 nM DAPI for 4 min. The cells were visualized using a Leica SP5 laser-scanning confocal microscope.

### Flow cytometry

The staining was performed at pH 6.0 or 8.0 throughout the procedure. HeLa-αFcRn-eGFP/β_2_m cells were harvested by trypsination and washed with PBS. 100 000 cells were used in each experiment and cells were fixed with 2% formaldehyde (Sigma Aldrich) in PBS for 10 min at r.t. Cells were thereafter washed with PBSC and resuspended in PBSC supplemented with 0.1% saponin (AppliChem, Darmstadt, Germany) and 770 nM human IgG-Alexa647 or HSA-Alexa647. The cells were incubated for 1 h at 8°C, washed with PBSC and resuspended in PBS supplemented with 1% Bovine serum albumin (Merck, Darmstadt, Germany). Cells were analyzed in a Gallios Flow Cytometer (Beckman Coulter, CA, USA) and 10000 events per sample were recorded. The data was processed using Kaluza software (Beckman Coulter).

## Results

### Vector design for expression of FcRn_ECD_

Genes encoding the extracellular domain of the α-chain of human FcRn (αFcRn_ECD_) and human β_2_m were inserted into the lentiviral transfer plasmids 2K7_bsd_ and 2K7_neo_. The genes were extended so that the resulting proteins would have an Ig-κ chain leader sequence in the N-terminus for export of the protein to the culture medium. In addition, αFcRn_ECD_ had a C-terminal linker followed by a FLAG-tag and β_2_m had a C-terminal linker followed by a His_6_-tag (Figure 1). The linkers were added to possibly allow better access to the tags. The lentiviral transfer plasmids also contained different resistance genes to allow for selection of cells where both constructs had been inserted.

### Protein Production and Purification

To insert the genes encoding αFcRn_ECD_ and β_2_m into the genome of SKOV-3 cells, the lentiviral transfer plasmids were packaged into non-replicating lentiviral particles, which were subsequently used for sequential transduction of the cells. The gene encoding human β_2_m was first inserted followed by the gene encoding αFcRn_ECD_, after which selection for double integrants was carried out. Following expansion of resistant cells, 1.5*10^7^ cells were plated in a multi-layered flask in complete medium to allow efficient attachment of the cells. After attachment, FcRn_ECD_ was produced in 560 ml medium lacking fetal bovine serum (FBS) since it was found that BSA is difficult to remove when affinity chromatography using immobilized IgG or IMAC is employed for purification (data not shown).

**Figure 2:**
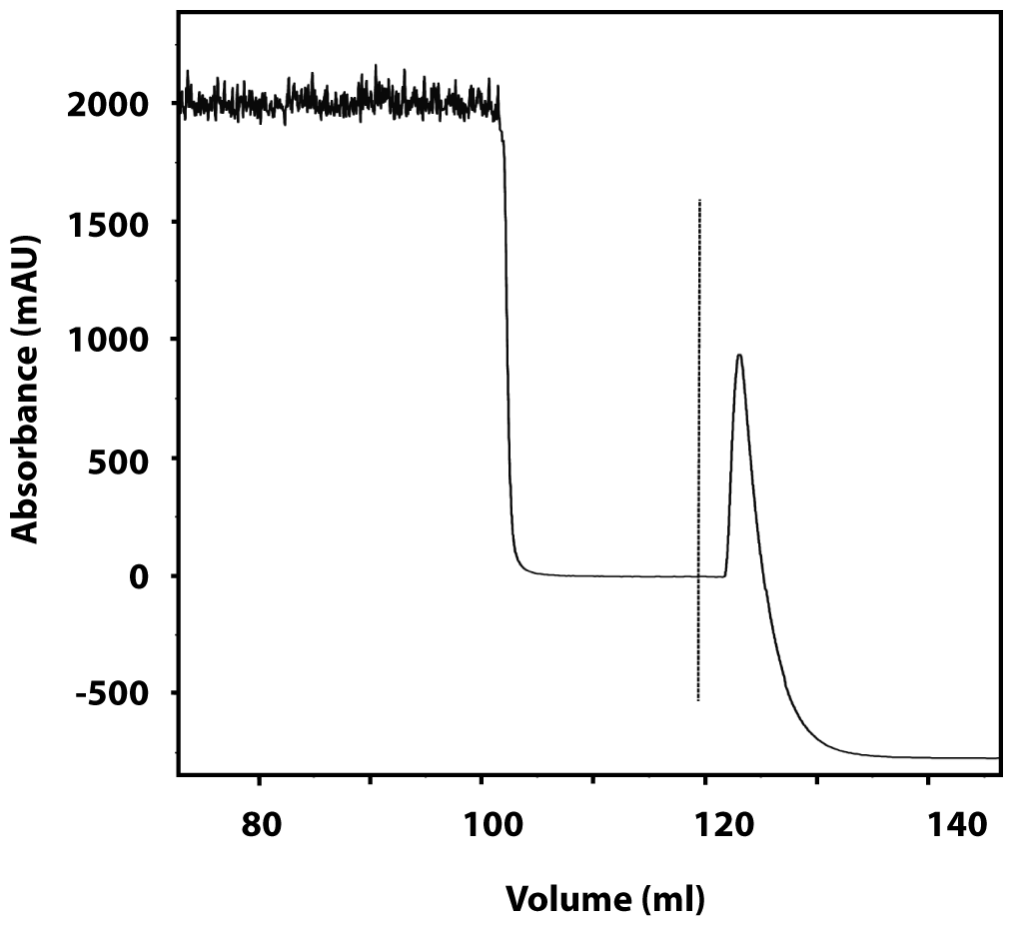
Representative chromatogram of the purification of FcRn_ECD_. Human FcRn_ECD_ with retained IgG binding was purified in a single affinity chromatography step on immobilized human IgG. The x-axis corresponds to the volume after sample injection on the column. The y-axis corresponds to measured absorbance. The dotted line corresponds to the start of elution where the pH is changed from 5.8 to 8.1.

Protein expression was performed for 120 h after which the medium was collected. To only capture FcRn_ECD_ with retained pH-dependent IgG binding, affinity chromatography using immobilized IgG was employed, where the column was loaded with supernatant at pH 5.8 and bound proteins were eluted at pH 8.1 (Figure 2). 

**Figure 3:**
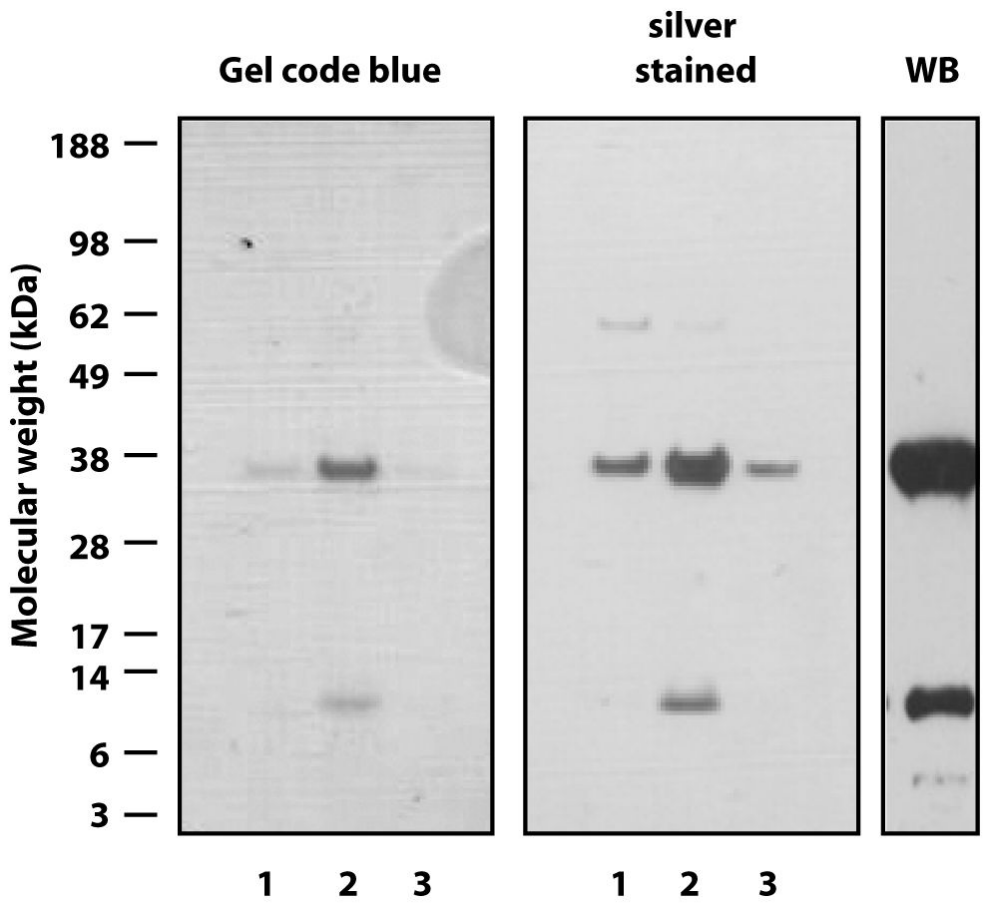
SDS-PAGE and Western blot analysis. Eluted fractions (1-3) from the representative purification of FcRn_ECD_ were analyzed by SDS-PAGE. The gel was stained with GelCode Blue Stain Reagent or was silver stained and showed essentially only two bands with a molecular mass of 36 and 12 kDa. WB: western blotting of pooled fractions using antibodies specific for FcRn and human β_2_m, identifying the protein bands as αFcRn_ECD_ and β_2_m.

To investigate the presence of two peptide chains with the expected molecular mass as well as to initially determine the purity of FcRn_ECD_, an SDS-PAGE analysis was performed on the eluted fractions (Figure 3). For the gel stained with Gel code blue, two bands were detected with a molecular weight of 12 and 36 kDa. This corresponds approximately with the theoretical molecular weights of the non-glycosylated peptide chains of 12 kDa for β_2_m and 31 kDa for αFcRn_ECD_. The αFcRn_ECD_ part of the protein contains one glycosylation site and it was therefore expected that its molecular mass should be higher than 31 kDa. For the silver stained gel an additional band around 66 kDa was detected in the first eluted fraction, which could correspond to BSA originating from cell attachment. The total amount of protein recovered in fraction 2 and 3 was pooled and corresponded to 1.4 mg/L culture medium. A western blot analysis on the pooled material using antibodies specific for FcRn and β_2_m showed essentially only the two major bands and in addition a very weak band below 12 kDa which could correspond to a degradation product, further indicating that the recovered protein was FcRn_ECD_. 

### Characterization of isolated FcRn_ECD_

**Figure 4:**
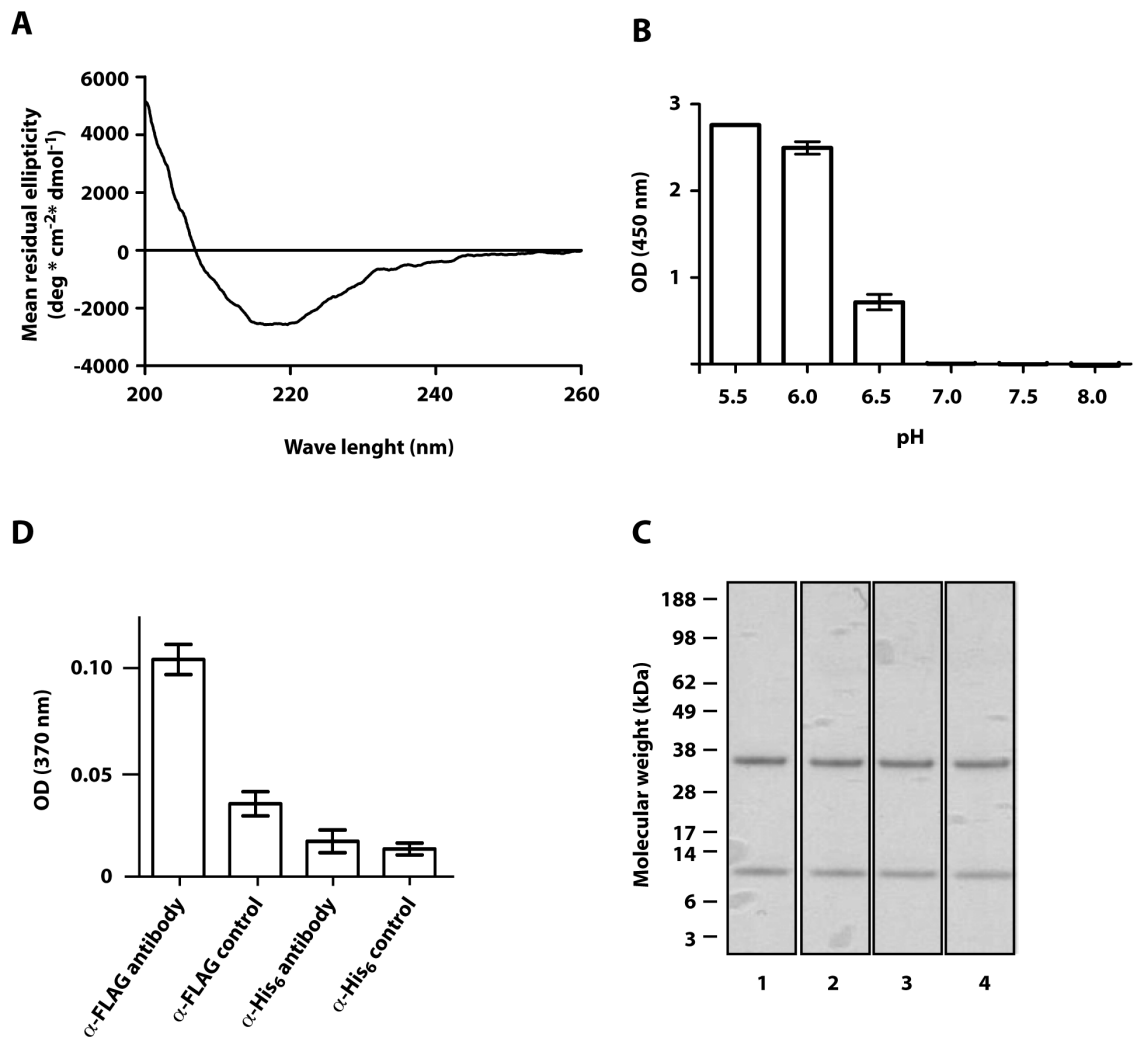
Characterization of FcRn_ECD_. (A) Representative spectrum of FcRn_ECD_ determined by circular dichroism spectroscopy. (B) The pH-dependent interaction between FcRn_ECD_ and IgG was investigated by ELISA. Immobilized FcRn_ECD_ was probed with human IgG at different pH values. Each data point is an average of three experiments with standard deviation. (C) The functionality of the FLAG-tag and the His_6_-tag was investigated by ELISA. Immobilized IgG was probed with FcRn_ECD_ followed by an α-FLAG antibody or an α-His_6_ antibody. In the control experiments FcRn_ECD_ was omitted. Each data point is an average of three experiments with standard deviation. (D) The tendency to precipitate or be degraded during storage was investigated by exposing FcRn_ECD_ to 8°C (Lane 1), -20°C (Lane 2), repeated cycles of freezing and thawing (Lane 3) or 37°C (Lane 4) during 48 h.

The secondary structure content of the purified material was investigated by circular dichroism spectroscopy to obtain an indication of its folding. The resulting spectrum is shown in Figure 4A and was similar to previously published spectra for FcRn_ECD_ [16,17]. The secondary structure content was calculated (Table 1) and a minor part was found to be helix whereas the majority was β-sheet and β-turn, similar to the content previously published by others [16]. In contrast to previous studies, the ratio between parallel and anti-parallel β-sheet was 1:1. Also, the amount of random coil was higher than previous studies. The difference compared to previous studies may partly be attributed to the addition of linkers and the FLAG- and His_6_-tags to our construct.

**Table 1:** Secondary structure content of FcRn_ECD_.

Structure	%
Helix	12.7
Anti-parallel	14.1
Parallel	14.1
Beta-turn	17.2
Random coil	42.1
Total	100.0

The pH-dependent interaction with IgG was analyzed by ELISA. Human IgG was immobilized followed by incubation with FcRn_ECD_ at different pH values. The results showed the expected pH-dependent binding [20], with interaction at pH 6.0 and below, which became gradually lower as the pH was increased and disappeared at pH 7.0 and above (Figure 4B). 

The functionality of the FLAG-tag and His_6_-tag was also analyzed by ELISA (Figure 4C). Immobilized IgG was probed with FcRn_ECD_ followed by an α-FLAG antibody or an α-His_6_ antibody. The results showed a significant difference between the sample and control when using the α-FLAG antibody, but no difference between the sample and control when using the α-His_6_ antibody. This indicates that the FLAG-tag may be used for IgG detection by ELISA whereas the His_6_-tag may not.

The tendency to precipitate or to be degraded was also investigated, where the material was incubated at 8°C, -20°C or 37°C for 48 h or was subjected to repeated cycles of freezing and thawing after which it was analyzed by SDS-PAGE. No apparent loss of material was detected at any of the temperatures tested or by repeated cycles of freezing and thawing (Figure 4D), indicating a stable protein.

**Figure 5:**
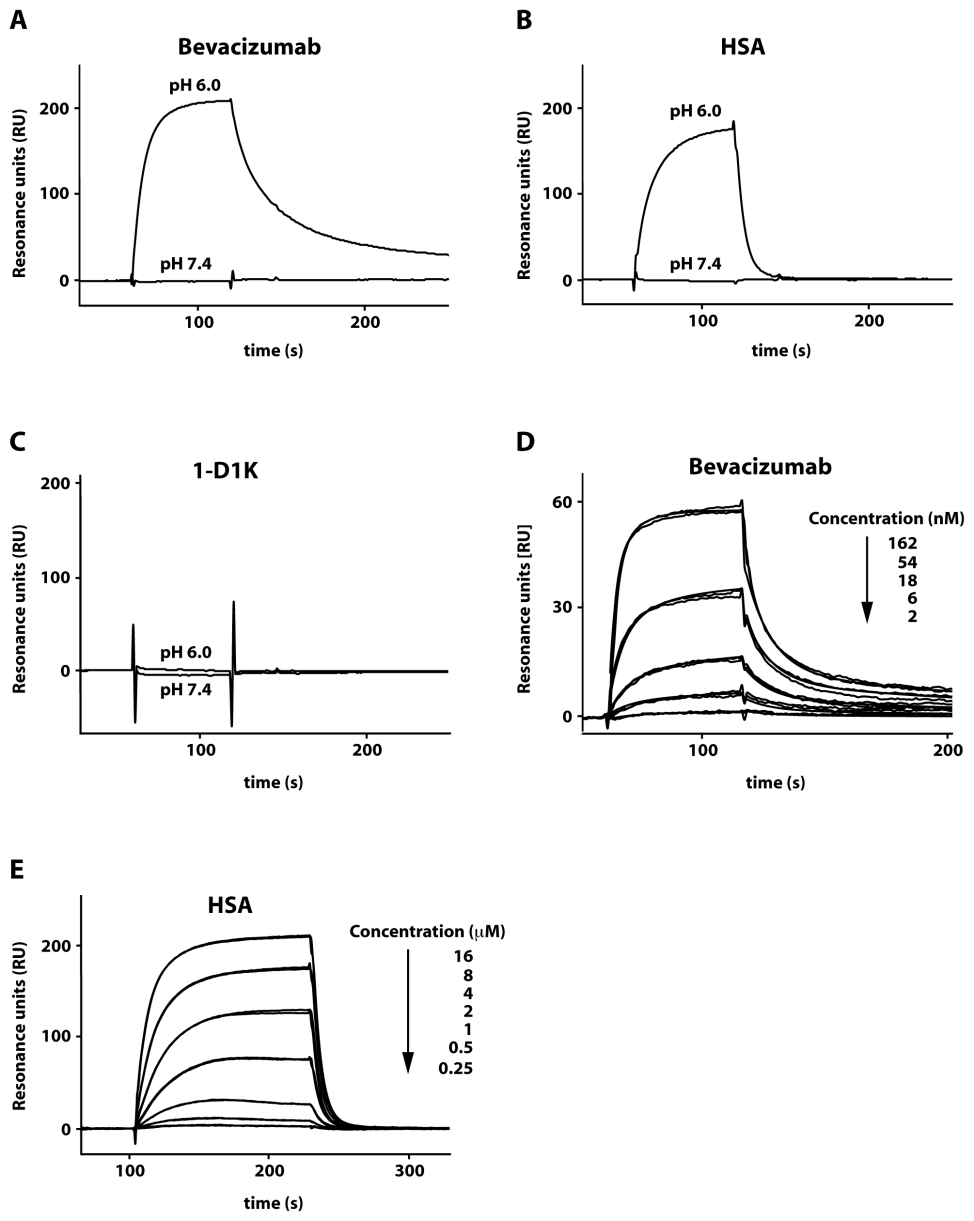
SPR analysis of ligands binding to FcRn_ECD_. Overlay of the sensorgram obtained after injection of 100 nM of Bevacizumab (A), 10 μM human SA (B) or 1 μM 1-D1K (C) at pH 6.0 or pH 7.4 over immobilized FcRn_ECD_. (D) Serial dilution of Bevacizumab injected over immobilized FcRn_ECD_ at pH 6.0. The sensorgram is an overlay of duplicate injections of each concentration and fitted curves obtained after evaluation. (E) Serial dilution of human SA injected over immobilized FcRn_ECD_ at pH 6.0. The sensorgram is an overlay of duplicate injections of each concentration.

Functional interaction between FcRn_ECD_ and IgG or HSA was further investigated by surface plasmon resonance (SPR) analysis. Injection of a human IgG1 (hIgG1) monoclonal antibody (Bevacizumab) at a concentration of 100 nM over a surface with immobilized FcRn_ECD_ resulted in reversible binding at pH 6.0 and no binding at pH 7.4 (Figure 5A). Similarly, injection of HSA over the same surface at a concentration of 10 μM resulted in reversible binding at pH 6.0 and no binding at pH 7.4 (Figure 5B). Injection of a mouse IgG1 (mIgG1) monoclonal antibody (1-D1K) at a concentration of 1 μM did not result in binding at pH 6.0 or 7.4. These results are in agreement with previous reports where hIgG1 and HSA show pH-dependent binding, but mIgG1 does not interact with human FcRn [16,33]. To determine the kinetic constants of the FcRn/hIgG1 interaction, a serial dilution of Bevacizumab was injected over immobilized FcRn (Figure 5D) at pH 6.0. This setup has previously been shown to give rise to complex binding kinetics and is often evaluated by the heterogenous ligand model [18,34]. The resulting kinetic constants are displayed in table 2. K_D1_ for the Bevacizumab/FcRn_EDC_ interaction was found to be similar to K_D1_ for a hIgG1/FcRn_EDC_ interaction previously determined using a mixture of human IgG1 [34]. By contrasts K_D2_ was 40 times lower than the value previously published [34]. Others have reported that the interaction between FcRn and different species of the same IgG subclass can differ [18]. The difference in K_D2_ could be the result of differences in the IgG1 analyte (Bevacizumab versus a mixture of human IgG1). The interaction between FcRn and HSA was also determined by injecting a serial dilution of HSA over immobilized FcRn_ECD_ at pH 6.0 (Figure 5E). From the equilibrium responses the K_D_ was estimated to 6.2 ± 0.0007 μM, which is in agreement with the previously reported affinity [9]. 

**Table 2:** Kinetic constants for the interaction between Bevacizumab and FcRn_ECD_.

K_a1_ (M^-1^s^-1^)	K_d1_ (s^-1^)	K_a2_ (M^-1^s^-1^)	K_d2_ (s^-1^)	K_D1_ (nM)	K_D2_ (nM)	(χ^2^)^a^
1.39*10^6^	0.0915	5.41*10^5^	0.00623	65	11	0.539

a statistical measure of the closeness of the fitted and the recorded curves.

### Expression and characterization of full-length human FcRn-eGFP fusion protein

The lentivirus based expression system was also used to create a cell line that stably over-expresses full-length FcRn as an N-terminal fusion to enhanced green fluorescent protein (eGFP). The gene encoding FcRn α-chain, including its native signal peptide, was sub-cloned into the lentiviral transfer plasmid pHR-cPPT-CMV-eGFP in frame with the gene encoding eGFP, already present in the vector (Figure 1C). The resulting plasmid and the plasmid 2K7_neo_-β_2_m was packaged into lentiviral particles and sequentially delivered to HeLa cells, which does not normally express detectable amounts of FcRn protein. 

**Figure 6:**
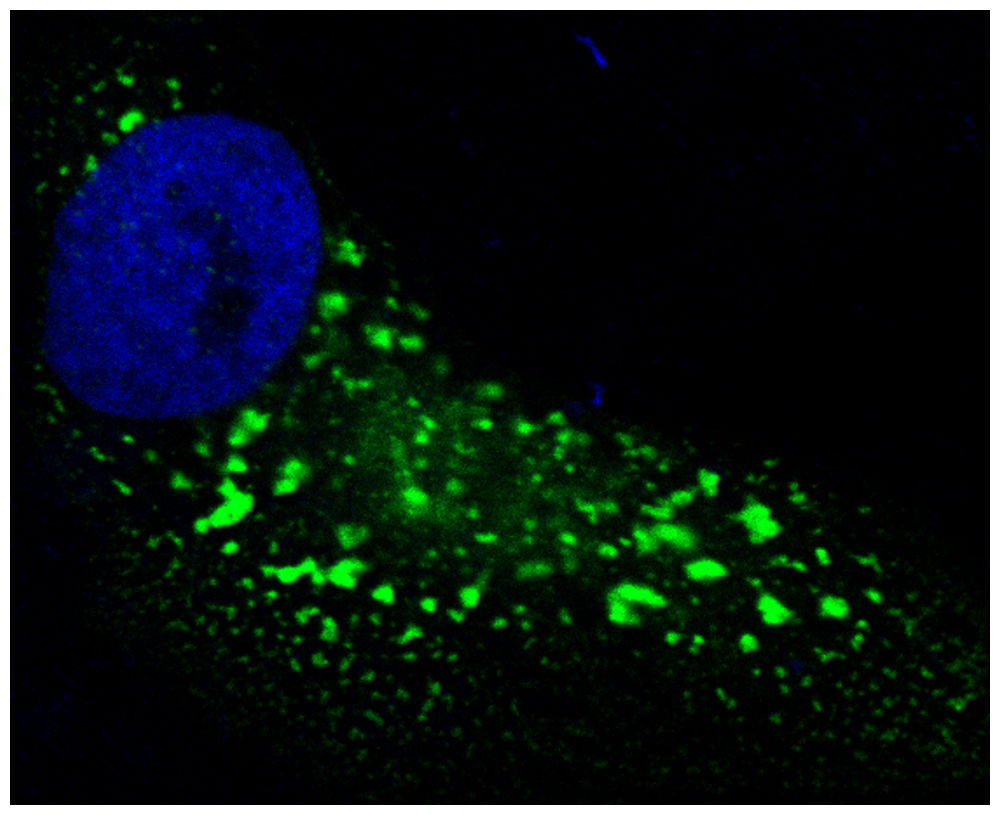
Image of a representative cell expressing human FcRn-eGFP. The cells were stained with DAPI (blue) and eGFP was colored green.

The cells were visualized by fluorescence microscopy (Figure 6). FcRn-eGFP is mainly localized intracellularly in discrete compartments, which is in agreement with previous studies which has been shown that FcRn is primarily localized in the endosomes [26].

**Figure 7:**
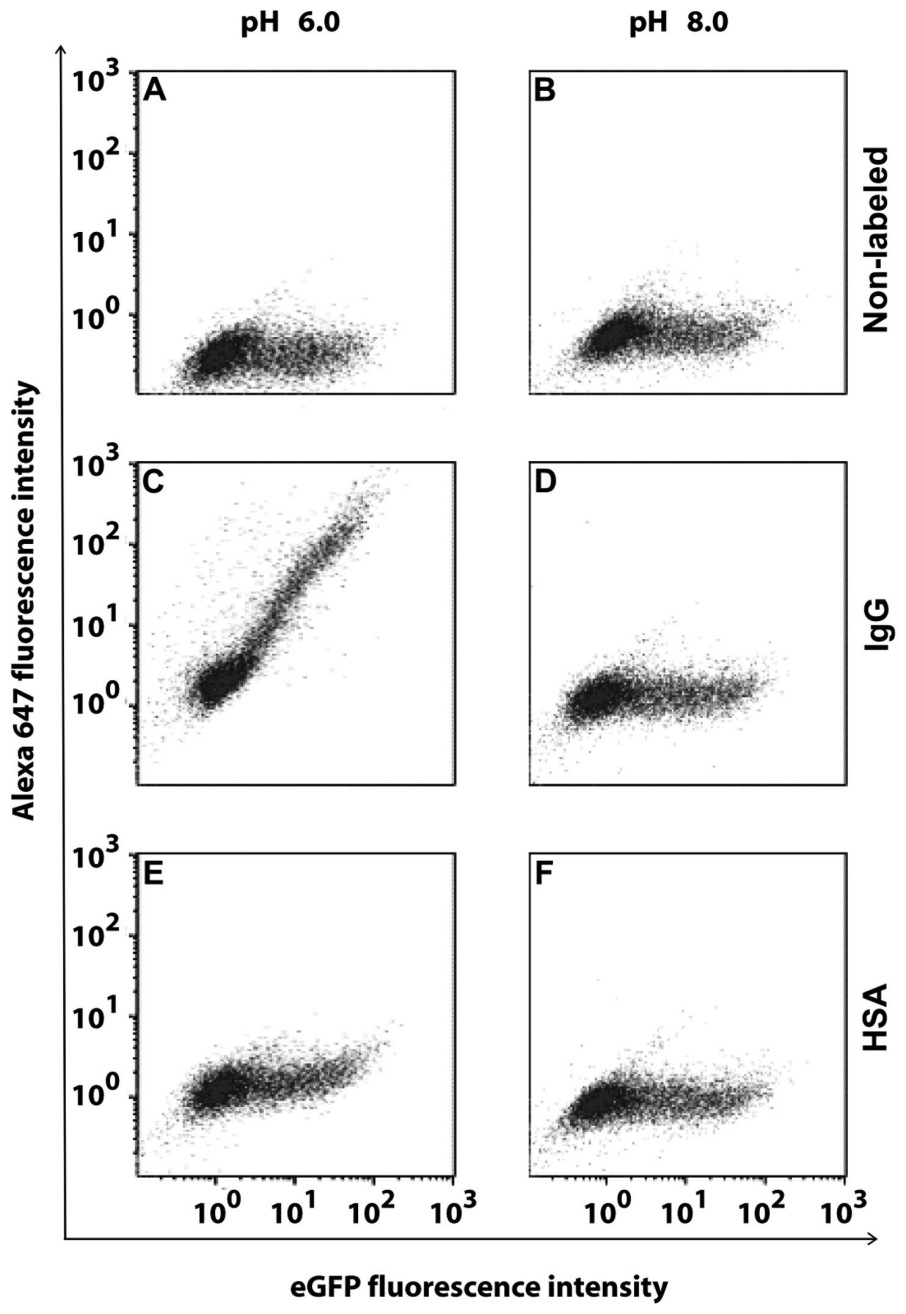
pH-dependent binding of human IgG or HSA to human FcRn-eGFP. HeLa cells over-expressing FcRn-eGFP was probed with human IgG or HSA. The eGFP fluorescence intensity of the cells was investigated (A, B). Cells were stained with human IgG labeled with Alexa647 (C, D) or HSA labeled with Alexa647 (E, F). Staining was performed at pH 6.0 (A, C, E) or pH 8.0 (B, D, F).

The cells were also permeabilized and probed with human IgG or HSA labeled with Alexa647 at pH 6.0 or pH 8.0 and analyzed by flow cytometry (Figure 7). Non-labeled cells display eGFP-fluorescence at both pH 6.0 and 8.0 that varies over several orders of magnitude showing that eGFP expression, and hence FcRn expression, varies considerably between individual cells (Figure 7A and 7B). Cells probed with IgG-Alexa647 at pH 6.0 display a linear relationship between eGFP-fluorescence and Alexa647 fluorescence, showing a linear correlation between the amounts of FcRn-eGFP expression and binding of human IgG-Alexa647. At pH 8.0, no increase in Alexa-647 fluorescence was found, showing that IgG was not able to bind to the cells at this pH. Cells probed with HSA-Alexa647 at pH 6.0 showed no increase in Alexa647 fluorescence as a function of eGFP-fluorescence, except for a slight increase for cells with the highest eGFP-fluorescence, showing that HSA was only able to interact with the cells that express the highest level of FcRn-eGFP (Figure 7E). This increase was lost at pH 8.0 showing that HSA cannot interact with the cells at that pH (Figure 7F). 

## Discussion

Studies on FcRn have revealed many important functions based on its interactions with IgG and HSA, including half-life extension of both proteins and trancytosis of IgG across the upper airway epitelium, the small proximal intestines and the placenta [12]. This knowledge has for example been used during development of therapeutic antibodies, which have their half-life modulated by interaction with FcRn [30]. Several engineering efforts have been conducted to increase the half-life of antibodies by increasing their affinity for FcRn [35,36]. For other applications a shorter half-life is desirable, which could potentially be obtained by decreasing the antibody’s affinity for FcRn. An example could be *in vivo* molecular imaging using radionuclide labeled antibodies, where a shorter serum half-life may result in higher target to blood ratio and hence improved contrast. In addition, development of novel routes of administration of protein-drugs has been investigated, such as pulmonary delivery of EPO fused to the Fc-part of IgG, where FcRn in the upper airway epitelium is trancytosing the drug to the blood stream [34]. To further refine strategies of this kind, *in vitro* investigation of the interaction between FcRn and the protein drug could lead to clues about its *in vivo* behavior. This in turn requires robust expression systems for FcRn in different formats, to provide plentiful sources of high quality receptor.

In a previous study on production of FcRn in a human derived cell line, transient transfection of plasmids encoding αFcRn_ECD_ and β_2_m were used [20]. Such expression systems are semi-stable and the expression cell line is limited to one that is expressing Epstein-Barr virus nuclear antigen 1 (EBNA-1) such as PEAK cells. In the present study, we have utilized a stable expression system, which is not limited to a particular cell line. The genes encoding αFcRn_ECD_ and β_2_m were stably inserted into the genome of the production host cell using a lentivirus derived gene delivery system pseudotyped with vesicular-stomatitis virus protein G. This gene delivery system can efficiently insert genes on chromosomes of a broad range of mammalian host cells, resulting in stable long term expression [37]. We chose the SKOV-3 cell line for FcRn production because of its human origin, ease to transduce with VSV-G pseudotyped lentivirus and also because it is fast growing and adheres well to culture plates even in serum free medium, which may be a problem with other cell lines [20]. 

For recombinant expression of FcRn_ECD_, a difficulty is that it is composed of two separate polypeptide chains that need to associate to form the full receptor, which means that two different plasmids needs to be used or both genes have to be combined on the same plasmid, either as two expression cassettes [17] or as a single bicistronic construct where the genes are separated by e. g. an internal ribosome binding site (IRES) [20]. By using two different lentiviral transfer vectors with different resistances, selection for cells with insertion of both genes was quick and easy.

FcRn_ECD_ was purified by a single affinity chromatographic step using immobilized IgG to only recover receptor with intact IgG-binding, yielding a protein of very high purity. In a similar study where the ECD of FcRn was produced in a human embryonic kidney-293 cell derivate (PEAK cells), IMAC purification was instead utilized, resulting in a protein of lower purity but with higher yield [20]. The lower yield in our study may partially be attributed to the use of serum free medium in contrast to the earlier study. The construct used in this study also contained a His_6_-tag, and is was possible to recover also our receptor by IMAC (data not shown). 

The pH-dependent binding to IgG was demonstrated in three different formats, where the receptor was captured on a column with immobilized IgG at pH 5.8 and eluted at pH 8.1, in ELISA and by SPR. The obtained results were similar to the results published in other studies [20,33,34]. 

FcRn-GFP has previously been used to study for example receptor traffic in cells, where transiently expressing cells were used [26]. In the present study, genes encoding β_2_m and the full-length FcRn α-chain as a fusion to eGFP were delivered using the lentivirus-derived system, which allowed for easy generation of stable cell lines. When cells were analyzed by flow cytometry a broad range of eGFP expression was found among the cells, corresponding to a variability in FcRn expression. Large differences in protein expression between individual cells have been shown in previous studies using lentiviral gene delivery systems, and an explanation could be that it is a consequence of the number of integrated gene copies and differences in the sites of integration in the genome [38]. By probing the cells with fluorescently labeled IgG at pH 6.0 a linear relationship between eGFP fluorescence and IgG-fluorescence was found, showing that more IgG can bind to the cells if they express more FcRn. For HSA the same pattern was found but less protein could bind to the cells. This result was not surprising since HSA has a lower affinity than IgG for FcRn [9]. For both IgG and HSA the binding to cells was lost when pH was increased to 8.0 demonstrating the pH-dependent binding also this format. Expression of FcRn as an eGFP-fusion opens up for studies of spatial localization of FcRn as well as co-localization with other fluorescent molecules in living cells by fluorescence microscopy, as well as binding studies using flow cytometry.

In conclusion, we have developed two expression systems for human FcRn, both as a truncated soluble protein and as a full-length membrane bound protein fusion to eGFP, utilizing lentivirus-derived vectors for chromosomal integration of the genes. Biochemical characterization of the soluble form showed a stable and highly pure protein with expected pH-dependent binding to IgG and HSA. The membrane bound form also showed an expected pH-dependent interaction with IgG and HSA. Using a lentivirus derived gene delivery system opens up for using a wide array of target cells to further elucidate FcRn function in tissues such as the blood brain barrier, lung epithelia and placenta. 
